# The Oxygen Equivalent of Lactate Accumulation and Sex: Similar Work–Lactate Slopes in Men and Women Regardless of Body or Fat‐Free Mass Scaling

**DOI:** 10.1096/fba.2025-00330

**Published:** 2026-03-17

**Authors:** Benedikt Meixner, Mascha Lenk, Billy Sperlich

**Affiliations:** ^1^ Department of Sport Science Julius‐Maximilians‐Universität Würzburg, Integrative and Experimental Exercise Science & Training Würzburg Germany; ^2^ Department of Sport Science and Sport Friedrich‐Alexander‐Universität Erlangen‐Nürnberg Erlangen Germany; ^3^ Iq‐Move Praxis Fraunberger Erlangen Germany

**Keywords:** carbohydrate metabolism, energy equivalent, glycolysis, lactate, sex differences

## Abstract

The oxygen equivalent of blood lactate accumulation (ΔLa) is commonly expressed relative to body mass (BM), yet BM includes adipose tissue which does not substantially contribute to glycolytic energy production nor lactate distribution space and may confound sex comparisons. We examined whether replacing BM with fat‐free mass (FFM) (i) improves the correspondence between calculated glycolytic work and 15‐s sprint work and (ii) attenuates sex differences in the work–lactate relationship. Seventy‐one trained cyclists (48 men, 23 women) performed a 15‐s all‐out seated cycling sprint on a Cyclus2 ergometer. Blood lactate was sampled at rest and repeatedly for 8 min post‐sprint; ΔLa was defined as peak minus pre‐exercise lactate. Glycolytic work (W_Gly_) was calculated using an oxygen‐equivalent approach with ΔLa scaled to either BM or FFM. General linear models included glycolytic work, sex, and 15‐s work. Men exhibited higher 15‐s work and ΔLa than women, but the slope of the relationship between W_Gly_ and 15‐s work did not differ by sex for either BM or FFM. Regression models using FFM explained slightly more variance in 15‐s work than BM (*R* ^2^ = 0.79 vs. 0.75). Adding sex improved model fit for both formulations (*R* ^2^ = 0.85 and 0.85, respectively), indicating primarily an intercept effect rather than a slope difference. Replacing BM with FFM provides only a small improvement in explaining 15‐s work and does not reveal sex‐specific differences in the work–lactate slope. Thus, the lactate oxygen equivalent appears sex‐invariant while FFM‐based scaling may still be preferred for a more physiologically grounded estimate of W_Gly_.

## Graphical abstract

Capillary blood lactate was sampled repeatedly after a 15‐s all‐out sprint in women (*n* = 23) and men (*n* = 48). Glycolytic energy contribution was estimated from lactate accumulation (ΔLa) scaled either to body mass or to fat‐free mass and related to 15‐s work. The relationship between mechanical work and glycolytic contribution is comparable between men and women, independent of whether ΔLa is scaled to body mass or fat‐free mass. Fat‐free mass scaling provides a physiologically more appropriate reference and slightly improves model fit.

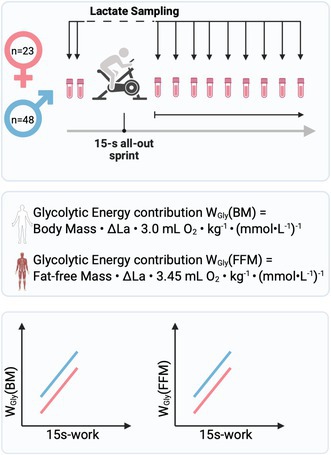

**Abbreviations**
BM: body mass
FFM: fat‐free mass
∆La: difference in lactate concentration from La_pre_ to La_peakpost_
La_maxpeakpost_
: Peak lactate concentration during the recovery period
La_pre_
: Mean value of two capillary blood lactate samples before sprint test
RPM: revolutions per minute
T1: First experimental visit
T2: Second experimental visit
W_Gly_
: Glycolytic Work

## 1 Introduction

Biological sex is associated with pronounced differences in exercise performance; however, a substantial proportion of these differences can be explained by body composition [1]. In particular, greater fat‐free mass (FFM) is strongly associated with performance and therefore represents a key covariate when comparing males and females [2, 3]. Across a broad range of physiological outcomes, including hemodynamics, substrate metabolism, and maximum oxygen uptake (V̇O_2_max), FFM is tightly coupled to function and capacity and should be considered a primary scaling variable in sex‐based comparisons [4, 5]. Traditionally, V̇O_2_max has been normalized to total body mass, an approach that fails to account for differences in body composition and consequently tends to exaggerate apparent sex differences in aerobic power [3]. Normalizing V̇O_2_max to fat‐free mass instead reduces these apparent differences and provides a more physiologically meaningful basis for interpretation in exercise physiology studies that treat sex as a biological variable [3]. Accordingly, many reported sex‐related differences in aerobic performance are more accurately attributable to differences in fitness and body composition rather than to sex per se [3] underscoring the importance of appropriate normalization for meaningful physiological comparisons.

However, the approach of interpreting physiological differences through appropriate normalization to metabolically relevant body compartments has largely been confined to aerobic outcomes, whereas anaerobic performance, particularly glycolytic power and capacity, has not been examined with the same rigor when sex differences are considered [6]. Because anaerobic energy turnover cannot be assessed directly, its quantification remains challenging [7, 8]. As a result, most analytical frameworks therefore rely on indirect estimates based on post‐exercise oxygen uptake and blood lactate accumulation to approximate total energy expenditure and the relative contributions of phosphagen, glycolytic, and oxidative pathways [9].

Seminal work by Margaria and colleagues introduced the concept of an oxygen equivalent of lactate accumulation, whereby a 1 mmol·L^−1^ increase in blood lactate concentration is assumed to correspond to approximately 2.7–3.3 mL O_2_·kg^−1^ body mass [7, 10, 11, 12]. This body‐mass–based normalization factor underpins the widely used three‐component model for partitioning energy system contributions across a range of exercise modes and experimental settings [9, 10, 13, 14, 15, 16], enabling the calculation of absolute glycolytic work (W_Gly_) [16].

However, by analogy with normalization issues identified for aerobic power [3], the continued reliance on total body mass in Margaria's oxygen‐equivalent framework may be problematic when comparing males and females. Although both fat mass and fat‐free mass contribute to total body mass, adipose tissue does not contribute to oxygen consumption during exercise and plays little role in lactate production, distribution, or removal during short, high‐intensity efforts because of its low water content [17, 18]. Modern body composition techniques now allow relatively precise estimation of fat‐free mass, and contemporary bioenergetic models explicitly recognize that lactate distributes within only a limited fraction of fat‐free mass rather than total body mass, necessitating normalization approaches that account for body composition [17, 19, 20]. Accordingly, defining glycolytic work as a function of blood lactate accumulation (ΔLa) scaled to body mass systematically overweights adipose tissue and may inflate apparent sex differences.

Supporting this concern, recent sprint‐cycling data show that glycolytic work calculated as ΔLa × fat‐free mass is strongly related to 15‐s mechanical work, with a similar relationship in males and females despite pronounced differences in body composition [17]. At the same time, experimental and modeling evidence indicates that reported sex differences in muscle fiber‐type distribution, muscle architecture, and peak lactate concentrations do not necessarily translate into differences in mechanical work performed per mmol of lactate, provided that active muscle mass is appropriately accounted for [17, 21, 22, 23, 24].

Taken together, these observations reveal a conceptual inconsistency in the treatment of sex differences across energy systems. If fat‐free mass is the appropriate scaling variable for V̇O_2_max when comparing males and females [3], then the oxygen equivalent of lactate accumulation should likewise be expressed relative to fat‐free mass rather than total body mass. This reformulation requires re‐scaling of the oxygen cost associated with a given increase in blood lactate concentration but would better align glycolytic energy estimates with the metabolically active tissue compartments involved in lactate production and distribution. Accordingly, a fat‐free‐mass–based scaling approach may provide a more physiologically grounded quantification of glycolytic energy contribution, enabling a more adequate comparison between sexes.

Therefore, the aim of the present study was to examine the relationship between blood lactate accumulation (ΔLa) and 15‐s sprint mechanical work (15 s‐work) when glycolytic energy contribution is quantified using the oxygen equivalent of lactate accumulation scaled either to total body mass (BM) or fat‐free mass (FFM). Specifically, we tested whether substituting FFM for BM in these calculations (i) strengthens the association between estimated glycolytic work and mechanical work and (ii) reduces or eliminates apparent sex differences in this relationship. We hypothesized that sex‐related differences in the ΔLa–work relationship would be largely resolved when glycolytic contribution is expressed relative to FFM rather than BM, thereby providing a physiological basis for advancing Margaria's original body‐mass–based equations toward an FFM‐based formulation. Importantly, this analysis does not aim to *re‐determine* the energy equivalent of lactate, which would require concurrent quantification of oxidative energy turnover and phosphagen contribution but rather addresses a foundational but often implicit assumption within the commonly used oxygen‐equivalent approach: which body compartment should be used when converting a concentration change in blood lactate (ΔLa, mmol·L^−1^) into an estimate of whole‐body glycolytic energy turnover?

## 2 Methods

The present analysis is a combined reanalysis of two previously published studies [17, 25]. Detailed testing protocols are described in the original publications. The datasets were combined because both studies included a baseline test performed after a standardized familiarization trial. After removal of duplicate participants tested at multiple time points, a total of 71 cyclists (48 males and 23 females) were included. A brief description of the testing procedures is presented below.

All participants were experienced road cyclists using clipless pedals and cycled regularly for exercise. Prior to the study, participants were informed of the study protocol and gave their written informed consent prior to participation. All procedures were approved by the Ethics Committee of Exercise Science and Training, Faculty of Human Sciences, University of Würzburg (EV2024/1‐1004) and conducted in accordance with the Declaration of Helsinki [26, 27].

### 2.1 Experimental Design

Only the second experimental visit to the laboratory was analyzed in this study. This visit was performed after a previous familiarization trial, which was completed at least 48 h earlier and within a period of 1 week.

All participants were instructed to keep a nutrition diary and to repeat their usual diet during the 24 h preceding each experimental visit [28]. Additionally, all participants were instructed to stay adequately hydrated, to eat a carbohydrate‐rich meal (i.e., a banana and a jam sandwich) at least 3 h before each visit, and to refrain from caffeine consumption on the day of testing.

During the first visit (familiarization, *n* = 50) or second visit (baseline testing, *n* = 23), body composition (i.e., fat‐free mass (FFM)) was assessed in all participants using eight‐electrode bioelectrical impedance analysis (InBody 720, Biospace, Des Moines, Iowa, USA).

All 15‐s all‐out cycle sprints were conducted on participants' personal road bikes installed on a Cyclus2 ergometer (RBM, Leipzig, Germany). The Cyclus2 is an electromagnetically braked ergometer and measures power with an accuracy error of 2% according to the manufacturer. All cyclists used their own shoes and pedals for all tests. For all three visits, all cyclists warmed up for 10 min of cycling at 1.5 W·kg^−1^ body mass and rested for 3 min [29].

The all‐out cycle sprint was performed in a seated position utilizing the large chainring (if applicable) of the participant's bike and the 15‐tooth cog of the ergometer. Data recording of the test started once a cadence of > 30 rpm was reached. The ergometer software was set to isokinetic mode at 130 rpm [29, 30, 31].

Capillary blood samples of the left earlobe were obtained twice during the resting period, once directly after the warm‐up while resting passively and once directly after the sprint, as well as every minute for 8 min after the 15‐s cycle sprint. Blood lactate concentration was determined using an amperometric–enzymatic method with a Biosen C‐Line analyzer (EKF Diagnostics, Barleben, Germany). Peak lactate concentration was taken as the highest measured value during the post‐exercise passive rest period. ΔLa was calculated as the difference between the mean of resting values (La_pre_) and the peak value attained during the post‐exercise resting period (La_maxpeakpost_).

### 2.2 Calculation of Glycolytic Work

The calculation of glycolytic energy contribution was based on previous work [11, 12]. An oxygen equivalent of 3 mL O_2_ per kg BM was assumed per 1 mmol·L^−1^ of accumulated lactate in capillary blood. To express the canonical Margaria‐type conversion (3.0 mL O_2_·kg^−1^·(mmol·L^−1^)^−1^) on an FFM basis, we applied a compartment substitution because ΔLa is a concentration change, estimating lactate‐equivalent energy requires multiplication by a reference mass. Accordingly, the BM‐based factor was re‐expressed for FFM by scaling with the individual BM/FFM ratio. For descriptive purposes, we also used the cohort‐level FFM‐based factor (~3.45 mL O_2_· kg FFM^−1^ ·(mmol·L^−1^)^−1^) corresponding to the average BM‐to‐FFM relationship in the present sample (Figure 3). This conversion does not constitute an independent physiological “re‐determination” thereof [32]; it is a re‐expression of the established BM‐based assumption on a different reference compartment. This oxygen equivalent was transformed to glycolytic work (W_Gly_) with the respective reference mass BM or FFM, assuming an energy equivalent of 21.1 kJ/L O_2_ and gross efficiency of 20% [33].

### 2.3 Statistical Analyses

Raw data was processed using Microsoft Excel. Statistical analyses (mean, standard deviations, and 95% confidence intervals) were computed with GraphPad Prism (10, Boston, MA, USA). Data normality for all measured variables (15 s‐work, ∆La, BM, FFM) was assessed using the Kolmogorov–Smirnov‐test and visual inspection, without requiring further transformation.

Uni‐ and multivariate regression analyses were performed to evaluate the strength of the relationship between relative and absolute 15 s‐work as dependent variable and covariates [2, 34]. Covariates included sex and W_Gly_(BM) or W_Gly_(FFM). Sex as a categorical parameter with 2 levels was transformed to a continuous parameter for inclusion in regression models, with females = 0 and males = 1.

## 3 Results

All mean ± SD values for raw data are displayed in Table 1. More detailed displays of the data can be found in the primary studies for this dataset [17, 25].

**TABLE 1:** Anthropometric and metabolic data of the participants during 15 s sprint cycling presented as Mean ± SD.

Parameter	Females	Males
*n*	23	48
BM (kg)	61.4 ± 6.3^a^	77.1 ± 9.6
FFM (kg)	50.8 ± 5.6^a^	67.8 ± 7.3
BF (%)	17.2 ± 4.2^a^	12.0 ± 4.1
ΔLa (mmol·L^−1^)	5.77 ± 1.29^a^	7.72 ± 2.21
W_Gly_(BM) (J)	5643 ± 1528^a^	9385 ± 2790
W_Gly_(FFM) (J)	5613 ± 1569^a^	9878 ± 2811
15s‐work (J)	8766 ± 1261^a^	13,469 ± 2160

Abbreviations: 15s‐work, mechanical work during the 15 s sprint; BF, body fat; BM, Body Mass; FFM, Fat‐free Mass; ΔLa, difference between pre‐ and peak post‐exercise lactate concentration.

^a^ Significant difference between male and female values.

**FIGURE 1:**
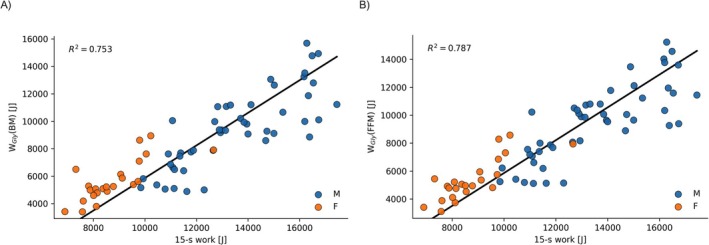
Scatter plots and overall regression line of 15 s‐work and glycolytic work calculated with (A) body mass and (B) fat‐free mass.

**FIGURE 2:**
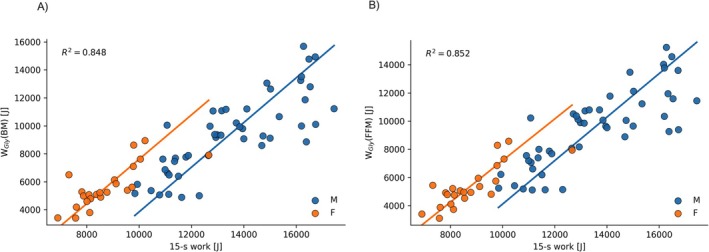
Scatter plots and sex‐specific regression line of 15 s‐work and glycolytic work calculated with (A) body mass and (B) fat‐free mass.

**FIGURE 3:**
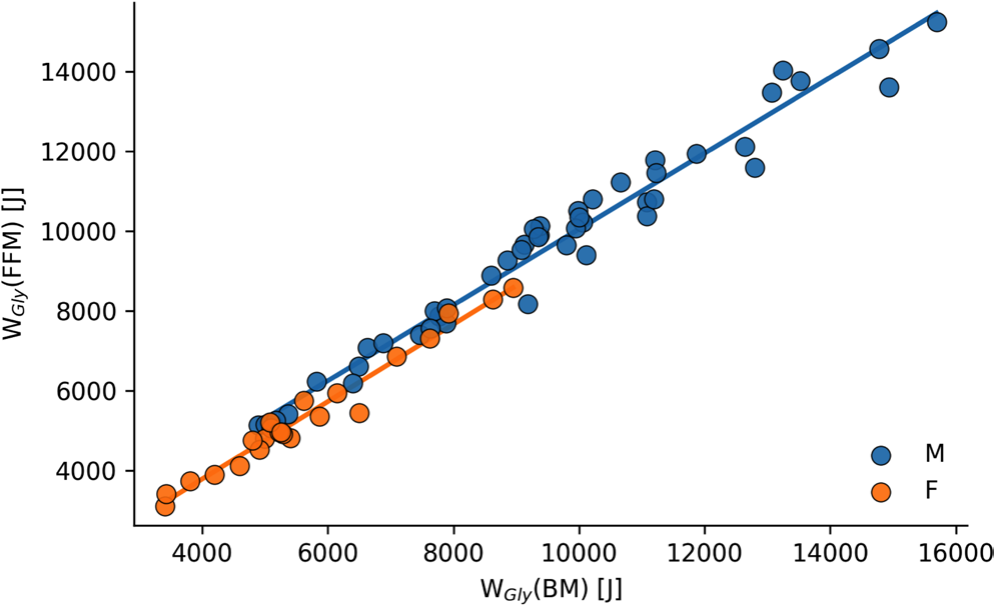
Scatter plot and sex‐specific regression line comparing glycolytic work calculated with body mass and fat‐free mass.

All regression models displayed no differences in slope for W_Gly_ between males and females and better *R* ^2^ with FFM‐based W_Gly_. Regression models improved significantly by including sex as a parameter, resulting in different intercepts of slopes (*p* < 0.01 for both BM and FFM as reference mass). No significant interaction of sex × W_Gly_ was found for either reference mass (BM: *p* = 0.98; FFM: *p* = 0.87). Results of the regression models are displayed in Table 2 and in Figures 1 and 2. VIF for W_Gly_ and sex was low in models for both regression models (1.52 (BM) resp. 1.66 (FFM)). The relation between W_Gly_(BM) and W_Gly_(FFM) is displayed in Figure 3.

**TABLE 2:** Results of all regression models to explain 15 s‐work; W_Gly_(BM) = body mass and W_Gly_(FFM) = fat‐free mass as factors in calculation of glycolytic work, sex coded as 1 = male and 0 = female.

Model	*R* ^2^	*B*		*p*		*β*		$\eta_{p}^{2}$	
		W_Gly_	Sex	W_Gly_	Sex	W_Gly_	Sex	W_Gly_	Sex
W_Gly_(BM)	0.753	0.84		< 0.001		0.87		0.75	
W_Gly_(FFM)	0.787	0.85		< 0.001		0.88		0.79	
W_Gly_(BM) + sex	0.848	0.63	2383	< 0.001	< 0.001	0.65	0.81	0.64	0.39
W_Gly_(FFM) + sex	0.852	0.68	220,639	< 0.001	< 0.001	0.71	0.68	0.65	0.31

When FFM is used instead of body mass in the calculation of glycolytic contribution, the estimated oxygen equivalent of lactate accumulation increases by approximately 1.15 mL O_2_ per mmol·L^−1^ΔLa.

When the canonical BM‐based conversion (3.0 mL O_2_·kg BM^−1^·(mmol·L^−1^)^−1^) is re‐scaled per kilogram of FFM, the implied conversion factor becomes 3.0 (BM/FFM). Using group means from Table 1, this yields an implied β of approximately 3.63 mL O_2_·kg FFM^−1^·(mmol·L^−1^)^−1^in females (61.4/50.8 × 3.0) and 3.41 mL O_2_·kg FFM^−1^·(mmol·L^−1^)^−1^ in males (77.1/67.8 × 3.0). This difference is a direct mathematical consequence of body‐composition differences (BM/FFM) rather than evidence for a sex‐specific energetic yield of lactate formation.

## 4 Discussion

The main findings of this study are that

- the slope of the relationship between estimated glycolytic work and mechanical power output does not differ between males and females, and
- substituting fat‐free mass for body mass in the calculation of glycolytic contribution results in only a marginal improvement in (R^2^).

From a physiological perspective, the modest improvement in model fit when substituting FFM for BM is not unexpected in trained cyclists, as variability in adiposity is limited and the BM‐to‐FFM correction is therefore small at the group level despite statistical significant differences in percent body fat. Under these conditions, the substitution primarily refines the physiological interpretation—by aligning the scaling compartment with metabolically relevant tissue—rather than substantially changing the explanatory power. Accordingly, we view the small Δ*R* ^2^ as consistent with an a priori expectation for an athletic cohort, while acknowledging that the practical impact of FFM‐based scaling may be greater in samples with wider variability in adiposity (e.g., recreational or clinical populations).

Thus, despite a clear physiological rationale for sex differences in anaerobic performance and body‐mass scaling, our data indicate that the relationship between mechanical work and metabolically estimated glycolytic work during a 15‐s all‐out sprint is similar in men and women, regardless of whether body mass or fat‐free mass is used as the referencing tissue mass in the oxygen‐equivalent calculation.

A useful interpretation our findings can be framed within the energetic perspective outlined by Ferretti and di Prampero [32]. They argue that during intense exercise the rate of energy release associated with glycolytic metabolism increases approximately linearly with mechanical power, as does the lactate accumulation rate. Accordingly, the relationship between glycolytic energy and lactate accumulation should be linear, with a relatively constant slope representing the energy equivalent of lactate accumulation. From this standpoint, our comparison of BM‐ versus FFM‐based scaling does not question the constancy of this equivalent. Rather, it addresses a methodological asymmetry: aerobic energy contribution is quantified in absolute terms, whereas ΔLa represents a concentration change and must be multiplied by a reference mass to obtain an absolute energetic estimate. If groups differ in body composition, BM‐based scaling may introduce systematic bias by including adipose tissue within the reference compartment, potentially producing apparent group differences that reflect scaling effects rather than physiological variation.

Using a subset of the present dataset, we have previously reported the relationship between 15‐s mechanical work and glycolytic contribution when expressed relative to fat‐free mass [17]. In contrast, other studies employing comparable 15‐s sprint protocols have calculated glycolytic contribution using body‐mass–based scaling factors in the tradition of Margaria [13, 16, 35], an approach that may be sufficiently accurate in cohorts that are relatively homogeneous with respect to body composition [36]. When sex is treated as a biological variable, however, body mass conflates metabolically active tissue with adipose tissue and may therefore represent a suboptimal reference. To our knowledge, no previous study has explicitly compared body‐mass and fat‐free‐mass–based formulations of the oxygen equivalent of lactate accumulation when examining sex differences in glycolytic work. The present findings extend the literature by demonstrating that fat‐free‐mass–based scaling yields only a modest improvement in model fit and does not alter the sex‐invariant slope of the work‐lactate relationship. Nevertheless, consistent with the rationale articulated earlier [3], we propose that relating glycolytic variables to fat‐free mass rather than body mass provides a more physiologically grounded and conceptually sex‐neutral framework for interpreting male–female comparisons. As a consequence, our FFM‐expressed value of 3.45 mL O_2_ per mmol·L^−1^ of lactate accumulation can be considered a starting point in trained cyclists but should be determined in different populations and exercise durations and modalities as well. It is limited by the fact that it is based on the original value of 3.0 mL O_2_ per mmol·L^−1^ and the body composition of our participants.

We assume that lactate generated during short, maximal sprints distributes primarily within metabolically active, water‐rich compartments of fat‐free mass [20] rather than across total body mass. This view is consistent with evidence showing minimal lactate accumulation in adipose tissue, its low water content, and the close coupling between body water and lactate distribution volume [18, 20, 37, 38].

If adipose tissue contributes little to lactate production, distribution, or removal, scaling glycolytic contribution to body mass inherently overweights metabolically less relevant tissue. Replacing body mass with fat‐free mass in the oxygen‐equivalent calculation thus constitutes a physiologically coherent refinement, even if it does not meaningfully change the slope of the work–lactate relationship in the present dataset.

Similar considerations apply to other small, water‐soluble metabolites whose primary distribution space and major sites of uptake reside in lean tissues. For example, whole‐body glucose disposal during exercise and in insulin‐stimulated conditions is largely driven by skeletal muscle, which scales with fat‐free mass rather than total body mass [39, 40]. Accordingly, many clamp and tracer studies express glucose uptake or insulin sensitivity per kilogram of fat‐free mass rather than per kilogram of body mass [41]. Adipose tissue is more directly involved in glucose handling than in lactate metabolism, functioning both as a site of glucose uptake and as an endocrine organ that secretes adipokines influencing whole‐body insulin sensitivity and hepatic glucose output. However, its contribution is primarily regulatory rather than limiting for dynamic, exercise‐related glucose fluxes [42]. Thus, using fat‐free mass or total body water, which is tightly linked to fat‐free mass, as the scaling variable is conceptually more consistent with the underlying anatomy and physiology than scaling to body mass when individuals differ substantially in adiposity [43, 44].

The prevailing calculation of glycolytic contribution as the product of body mass and blood lactate accumulation (ΔLa), multiplied by a constant oxygen equivalent, thus represents a pragmatic but relatively coarse approximation. When a substantial proportion of body mass consists of metabolically less active adipose tissue, this formulation will systematically bias estimates of glycolytic work, particularly when individuals or groups differ in body fat percentage. Replacing body mass with fat‐free mass within the same conceptual framework does not alter the underlying logic of Margaria's approach but instead shifts the scaling to a tissue compartment more directly involved in lactate production, distribution, and removal. In the present dataset, this adjustment required an increase of the conversion factor by approximately 20% to preserve the correspondence between estimated glycolytic work and observed 15‐s mechanical work. Importantly, this numerical rescaling did not affect the sex‐invariant slope of the work–lactate relationship, indicating that the choice of reference mass influences the magnitude of estimated glycolytic contribution without introducing or obscuring a sex effect in this specific context.

Recently, a model was proposed that incorporates sex‐specific lactate equivalent values to account for differences in body composition between male and female sprinters [36]. At the group level, this approach assumes a higher proportion of body fat in females and, on this basis, applies a lower oxygen equivalent of lactate accumulation for women. The authors justify this adjustment using similar physiological arguments, namely that adipose tissue contributes minimally to lactate production and distribution.

However, a more sex‐neutral way to represent this concept is to assume an identical glycolytic yield (i.e., a common oxygen‐equivalent conversion factor per mmol·L^−1^ ΔLa) when expressed relative to fat‐free mass, and to relate this value to body mass only at a secondary level, particularly in sporting contexts where relative performance is decisive. Contrary to expectation, our data show no sex differences in the oxygen equivalent of lactate accumulation, irrespective of whether body mass or fat‐free mass is used as the reference.

Notably, observed differences in 15‐s mechanical work between males and females are attributable to a vertical offset between groups rather than to differences in slope. This offset is fully accounted for in models that include fat‐free mass as the reference tissue, whereas it remains unexplained when body mass is used instead (see Supplementary Files).

Previous work has suggested that individual slopes relating glycolytic work to lactate accumulation may vary [45]. Using methodological approaches that differ from the classical framework proposed by Margaria, reported oxygen equivalents of lactate accumulation have ranged from approximately 2.6 to 4.0 mL O_2_·(mmol·L^−1^ΔLa)^−1^ [45]. However, part of this relatively wide range in estimated glycolytic yield may reflect differences in scaling methodology rather than true physiological variability and may be reduced when fat‐free mass is used as the reference compartment instead of total body mass.

Consistent with prior findings in aerobic fitness [3], we argue that analyses of anaerobic glycolytic performance should likewise relate metabolic variables to fat‐free mass rather than body mass when examining differences between males and females. While further refinement of the oxygen equivalent, particularly by accounting for lactate distribution and elimination processes, represents an important avenue for future research, these processes are also more closely linked to fat‐free mass than to total body mass. Given that lactate elimination depends on cardiorespiratory function and oxygen uptake, appropriate scaling of these processes should therefore normalize to fat‐free mass as well [3].

Future work is required to refine the correction factor associated with the classical oxygen equivalent of lactate accumulation (2.7–3.3 mL O_2_·kg^−1^ body mass per mmol·L^−1^ΔLa), given that fat‐free mass represents a smaller compartment than total body mass. In the present dataset, this adjustment amounted to an increase of approximately 1.15‐fold; however, this value should be interpreted with caution, as it was derived from a single sprint duration and a relatively homogeneous cohort of trained athletes. Moreover, the magnitude of this factor is likely influenced by the method used to assess body composition, since estimates of fat‐free mass vary depending on the measurement technique employed [46].

At a physiological level, however, there is currently little evidence to support the assumption of sex‐specific differences in the efficiency of lactate production, even when accounting for variation in muscle fiber‐type distribution or muscle composition [23].

Body‐composition scaling is only one contributor to uncertainty in lactate‐based estimates of glycolytic energy turnover [32]. Even within an energetic framework that treats lactate accumulation as a proxy for anaerobic glycolysis, additional variability arises from (i) inter‐individual differences in lactate distribution volume and mixing kinetics, (ii) lactate oxidation and shuttling during and shortly after exercise, and (iii) the biochemical coupling between lactate formation and ATP turnover (often expressed as the phosphate/lactate ratio), which may deviate modestly from theoretical assumptions under physiological conditions.

In this context, the present work does not claim that FFM‐based scaling resolves all error sources; rather, it targets a *structural* inconsistency—multiplying a concentration change by a mass compartment that includes metabolically less relevant adipose tissue—thereby improving conceptual coherence when groups differ in adiposity.

## 5 Strengths and Limitations

A major strength of the present analysis is the use of two independent datasets employing comparable sprint protocols, which allowed robust examination of the work–lactate relationship across a relatively large sample. In addition, sex‐specific regression analyses demonstrated comparable goodness of fit, supporting the consistency of the observed relationships within males and females.

Nevertheless, because the datasets were combined, equal numbers of male and female participants could not be achieved, potentially influencing the combined regression models through the greater statistical weight of the larger male sample. Fat‐free mass was estimated using bioelectrical impedance analysis, which may also have influenced the results, as no gold‐standard body‐composition technique was available. Differences in assessment methods could affect not only the accuracy of fat‐free mass estimation but also the derived correction factor for the oxygen equivalent of lactate accumulation. Finally, the present study examined a single sprint duration and exercise modality and did not directly characterize lactate kinetics or hormonal status. Future studies should therefore include sex‐matched samples, employ reference‐standard body‐composition methods, and extend this framework to different exercise tasks, sprint durations, and body‐composition profiles.

## 6 Conclusion

Based on the present data in trained cyclists we conclude the ratio of mechanical work output to estimated glycolytic contribution is comparable between men and women, regardless of whether scaling is performed using BM or fat‐free mass FFM. Although FFM‐based normalization is physiologically more appropriate and yields a modest improvement in model fit, it does not uncover sex‐specific differences in the work–lactate relationship. Instead, this approach primarily facilitates a more sex‐neutral interpretation of glycolytic efficiency, particularly in study populations exhibiting greater variability in adiposity.

## Author Contributions

B.M.: conceptualization, data curation, investigation, methodology, formal analysis, project administration, visualization, writing – original draft; B.S.: methodology, formal analysis, project administration, supervision, validation, writing – review and editing; M.L.: writing – review and editing.

## Funding

The authors have nothing to report.

## Conflicts of Interest

The authors declare no conflicts of interest.

## Supporting information


**Data S1:** fba270097‐sup‐0001‐DataS1.docx.

## Data Availability

The data that support the findings of this study are available on reasonable request from the corresponding author. The data are not publicly available due to privacy or ethical restrictions.

## References

[1] B. Meixner , M. Schaffarczyk , and B. Sperlich , “Method Modulates: Protocol Choices Shape Sex Differences in the Determination of Exercise Intensity,” American Journal of Physiology. Regulatory, Integrative and Comparative Physiology 330 (2026): R174–R182, 10.1152/ajpregu.00266.2025. 41525307

[2] J. Makovey , V. Naganathan , and P. Sambrook , “Gender Differences in Relationships Between Body Composition Components, Their Distribution and Bone Mineral Density: A Cross‐Sectional Opposite Sex Twin Study,” Osteoporosis International 16, no. 12 (2005): 1495–1505, 10.1007/s00198-005-1841-4. 15838718

[3] T. R. Tripp , H. Kontro , J. B. Gillen , and M. J. MacInnis , “Fit for Comparison: Controlling for Cardiorespiratory Fitness in Exercise Physiology Studies of Sex as a Biological Variable,” Journal of Physiology 603, no. 8 (2025): 2219–2230, 10.1113/JP287735. 40120131 PMC12013801

[4] M. J. Joyner , “Physiological Limits to Endurance Exercise Performance: Influence of Sex,” Journal of Physiology 595, no. 9 (2017): 2949–2954, 10.1113/JP272268. 28028816 PMC5407964

[5] Ø. Skattebo , M. Martin‐Rincon , B. Rud , et al., “Determinants of Maximal Oxygen Uptake in Highly Trained Females and Males: A Mechanistic Study of Sex Differences Using Advanced Invasive Methods,” Journal of Physiology (2025). Online ahead of print, 10.1113/JP289218. 40974561

[6] O. J. Quittmann , “Maximal Lactate Accumulation Rate (˙cLamax): Current Evidence and Future Directions for Exercise Testing and Training,” European Journal of Applied Physiology 126 (2025): 1–36, 10.1007/s00421-025-06022-7. 41171430 PMC12881007

[7] P. E. di Prampero and G. Ferretti , “The Energetics of Anaerobic Muscle Metabolism: A Reappraisal of Older and Recent Concepts,” Respiration Physiology 118, no. 2 (1999): 103–115, 10.1016/S0034-5687(99)00083-3. 10647856

[8] P. B. Gastin , “Energy System Interaction and Relative Contribution During Maximal Exercise,” Sports Medicine 31, no. 10 (2001): 725–741, 10.2165/00007256-200131100-00003. 11547894

[9] J. Brochhagen and M. W. Hoppe , “Validation of the Metabolic Power Model During Three Intermittent Running‐Based Exercises With Emphasis on Aerobic and Anaerobic Energy Supply,” Frontiers in Sports and Active Living 7 (2025): 1583313, 10.3389/fspor.2025.1583313. 40313786 PMC12043615

[10] R. Beneke , C. Pollmann , I. Bleif , R. M. Leithäuser , and M. Hütler , “How Anaerobic Is the Wingate Anaerobic Test for Humans?,” European Journal of Applied Physiology 87, no. 4–5 (2002): 388–392, 10.1007/s00421-002-0622-4. 12172878

[11] R. Margaria , P. Cerretelli , P. E. Diprampero , C. Massari , and G. Torelli , “Kinetics and Mechanism of Oxygen Debt Contraction in Man,” Journal of Applied Physiology 18 (1963): 371–377, 10.1152/jappl.1963.18.2.371. 13932994

[12] R. Margaria , P. Cerretelli , and F. Mangili , “Balance and Kinetics of Anaerobic Energy Release During Strenuous Exercise in Man,” Journal of Applied Physiology 19 (1964): 623–628, 10.1152/jappl.1964.19.4.623. 14195570

[13] D. Archacki , J. Zieliński , B. Pospieszna , M. Włodarczyk , and K. Kusy , “The Contribution of Energy Systems During 15‐Second Sprint Exercise in Athletes of Different Sports Specializations,” PeerJ 12 (2024): e17863, 10.7717/peerj.17863. 39193515 PMC11348913

[14] J. O. Langley , S. C. Ng , E. E. Todd , and M. S. Porter , “V˙ La(Max): Determining the Optimal Test Duration for Maximal Lactate Formation Rate During All‐Out Sprint Cycle Ergometry,” European Journal of Applied Physiology 124 (2024): 2461–2472, 10.1007/s00421-024-05456-9. 38555335

[15] F. Milioni , J. V. M. Leite , R. Beneke , R. A. B. de Poli , M. Papoti , and A. M. Zagatto , “Table Tennis Playing Styles Require Specific Energy Systems Demands,” PLoS One 13, no. 7 (2018): e0199985, 10.1371/journal.pone.0199985. 30020946 PMC6051612

[16] W.‐H. Yang , S.‐Y. Park , T. Kim , H.‐J. Jeon , O. Heine , and S. Gehlert , “A Modified Formula Using Energy System Contributions to Calculate Pure Maximal Rate of Lactate Accumulation During a Maximal Sprint Cycling Test,” Frontiers in Physiology 14 (2023): 1147321, 10.3389/fphys.2023.1147321. 37123252 PMC10133696

[17] B. J. Meixner , V. Nusser , K. Koehler , M. Sablain , J. Boone , and B. Sperlich , “Relationship of Peak Capillary Blood Lactate Accumulation and Body Composition in Determining the Mechanical Energy Equivalent of Lactate During Sprint Cycling,” European Journal of Applied Physiology 124, no. 11 (2024): 3399–3407, 10.1007/s00421-024-05529-9. 38951183 PMC11519294

[18] T. Woodcock , “Fluid Physiology Part 1: Volume and Distribution of Water and Its Major Solutes Between Plasma, the Interstitium and Intracellular Fluid,” in Rational Use of Intravenous Fluids in Critically Ill Patients, ed. M. L. N. G. Malbrain , A. Wong , P. Nasa , and S. Ghosh (Springer International Publishing, 2024), 47–74, 10.1007/978-3-031-42205-8_2.

[19] F. A. Engel , B. Sperlich , C. Stockinger , S. Härtel , K. Bös , and H.‐C. Holmberg , “The Kinetics of Blood Lactate in Boys During and Following a Single and Repeated All‐Out Sprints of Cycling Are Different Than in Men,” Applied Physiology, Nutrition, and Metabolism 40, no. 6 (2015): 623–631, 10.1139/apnm-2014-0370. 25942632

[20] A. Mader , “Glycolysis and Oxidative Phosphorylation as a Function of Cytosolic Phosphorylation State and Power Output of the Muscle Cell,” European Journal of Applied Physiology 88, no. 4–5 (2003): 317–338, 10.1007/s00421-002-0676-3. 12527960

[21] C. Bailleul , N. Hodson , S. Abou Sawan , D. Kumbhare , D. R. Moore , and J. B. Gillen , “The Influence of Sex on Fiber‐Specific Indices of Oxidative Capacity in Human Skeletal Muscle,” American Journal of Physiology. Regulatory, Integrative and Comparative Physiology 329, no. 1 (2025): R70–R80, 10.1152/ajpregu.00298.2024. 40408260

[22] M. Esbjörnsson‐Liljedahl , K. Bodin , and E. Jansson , “Smaller Muscle ATP Reduction in Women Than in Men by Repeated Bouts of Sprint Exercise,” Journal of Applied Physiology 93, no. 3 (2002): 1075–1083, 10.1152/japplphysiol.00732.1999. 12183505

[23] Z.‐H. He , R. Bottinelli , M. A. Pellegrino , M. A. Ferenczi , and C. Reggiani , “ATP Consumption and Efficiency of Human Single Muscle Fibers With Different Myosin Isoform Composition,” Biophysical Journal 79, no. 2 (2000): 945–961, 10.1016/S0006-3495(00)76349-1. 10920025 PMC1300991

[24] B. Meixner , M. Matzka , and B. Sperlich , “Comparison of Maximal Glycolytic Rate From Ergometer to On‐Water Sprinting in Elite Canoe Polo Players,” Applied Physiology, Nutrition, and Metabolism 50 (2025): 1–10, 10.1139/apnm-2024-0450. 40138709

[25] B. Meixner , J. Stegmaier , P. Renner , K. Koehler , W. H. Yang , and B. Sperlich , “Supplementation of Creatine Monohydrate Improves Sprint Performance but Has no Effect on Glycolytic Contribution: A Nonrandomized, Placebo‐Controlled Crossover Trial in Trained Cyclists,” Current Developments in Nutrition 9, no. 2 (2025): 104561, 10.1016/j.cdnut.2025.104561. 40041626 PMC11876829

[26] D. J. Harriss and G. Atkinson , “International Journal of Sports Medicine ‐ Ethical Standards in Sport and Exercise Science Research,” International Journal of Sports Medicine 30, no. 10 (2009): 701–702, 10.1055/s-0029-1237378. 19809942

[27] World Medical Association , “World Medical Association Declaration of Helsinki: Ethical Principles for Medical Research Involving Human Subjects,” JAMA 310, no. 20 (2013): 2191–2194, 10.1001/jama.2013.281053. 24141714

[28] N. A. Jeacocke and L. M. Burke , “Methods to Standardize Dietary Intake Before Performance Testing,” International Journal of Sport Nutrition and Exercise Metabolism 20, no. 2 (2010): 87–103, 10.1123/ijsnem.20.2.87. 20479482

[29] O. J. Quittmann , Y. M. Schwarz , J. Mester , T. Foitschik , T. Abel , and H. K. Strüder , “Maximal Lactate Accumulation Rate in All‐Out Exercise Differs Between Cycling and Running,” International Journal of Sports Medicine 42, no. 4 (2021): 314–322, 10.1055/a-1273-7589. 33137832

[30] J. Adam , M. Ohmichen , E. Ohmichen , et al., “Reliability of the Calculated Maximal Lactate Steady State in Amateur Cyclists,” Biology of Sport 32, no. 2 (2015): 97–102, 10.5604/20831862.1134311. 26028808 PMC4296210

[31] N. Nitzsche , L. Baumgärtel , and H. Schulz , “Comparison of Maximum Lactate Formation Rates in Ergometer Sprint and Maximum Strength Loads,” Deutsche Zeitschrift fur Sportmedizin 69, no. 1 (2018): 13–18, 10.5960/dzsm.2017.312.

[32] G. Ferretti and P. E. di Prampero , “A Reassessment of the Energetic Significance of Blood Lactate Accumulation During Exercise,” European Journal of Applied Physiology (2026). Online ahead of print, 10.1007/s00421-026-06134-8. PMC13013375 41677846

[33] C. B. Scott , “Contribution of Anaerobic Energy Expenditure to Whole Body Thermogenesis,” Nutrition & Metabolism 2, no. 1 (2005): 14, 10.1186/1743-7075-2-14. 15958171 PMC1182393

[34] A. D. England , S. Musigwa , A. Kumar , et al., “Sex Proportion as a Covariate Increases the Statistical Test Power in Growth Performance Based Experiments Using As‐Hatched Broilers,” PLoS One 18, no. 1 (2023): e0280040, 10.1371/journal.pone.0280040. 36662683 PMC9857968

[35] D. Archacki , J. Zieliński , M. Ciekot‐Sołtysiak , E. A. Zarębska , and K. Kusy , “Sex Differences in the Energy System Contribution During Sprint Exercise in Speed‐Power and Endurance Athletes,” Journal of Clinical Medicine 13, no. 16 (2024): 4812. 39200953 10.3390/jcm13164812 PMC11355823

[36] J. Briand , P. E. di Prampero , C. Osgnach , G. Thibault , and J. Tremblay , “Quantifying Metabolic Energy Contributions in Sprint Running: A Novel Bioenergetic Model,” European Journal of Applied Physiology 125 (2025): 3521–3541, 10.1007/s00421-025-05831-0. 40536521 PMC12678604

[37] J. Grip , T. Falkenström , P. Promsin , J. Wernerman , Å. Norberg , and O. Rooyackers , “Lactate Kinetics in ICU Patients Using a Bolus of (13)C‐Labeled Lactate,” Critical Care 24, no. 1 (2020): 46, 10.1186/s13054-020-2753-6. 32041652 PMC7011254

[38] E. Hagström‐Toft , S. Enoksson , E. Moberg , J. Bolinder , and P. Arner , “Absolute Concentrations of Glycerol and Lactate in Human Skeletal Muscle, Adipose Tissue, and Blood,” American Journal of Physiology 273, no. 3 Pt 1 (1997): E584–E592, 10.1152/ajpendo.1997.273.3.E584. 9316449

[39] C. J. Rebello , D. Zhang , J. P. Kirwan , et al., “Effect of Exercise Training on Insulin‐Stimulated Glucose Disposal: A Systematic Review and Meta‐Analysis of Randomized Controlled Trials,” International Journal of Obesity 47, no. 5 (2023): 348–357. 36828899 10.1038/s41366-023-01283-8 PMC10148910

[40] L. Wu , F. Chen , J. Liu , et al., “The Relationship Between Fat‐Free Mass and Glucose Metabolism in Children and Adolescents: A Systematic Review and Meta‐Analysis,” Frontiers in Pediatrics 10 (2022): 864904. 35558370 10.3389/fped.2022.864904 PMC9087035

[41] E. Ferrannini , P. Iozzo , K. A. Virtanen , M.‐J. Honka , M. Bucci , and P. Nuutila , “Adipose Tissue and Skeletal Muscle Insulin‐Mediated Glucose Uptake in Insulin Resistance: Role of Blood Flow and Diabetes,” American Journal of Clinical Nutrition 108, no. 4 (2018): 749–758. 30239554 10.1093/ajcn/nqy162

[42] G. Sancar and A. L. Birkenfeld , “The Role of Adipose Tissue Dysfunction in Hepatic Insulin Resistance and T2D,” Journal of Endocrinology 262, no. 3 (2024): e240115. 38967989 10.1530/JOE-24-0115 PMC11378142

[43] S. Liu , Y. Yang , J. Song , et al., “Total Body Water/Fat‐Free Mass Ratio as a Valuable Predictive Parameter for Mortality in Maintenance Hemodialysis Patients,” Medicine 101, no. 31 (2022): e29904, 10.1097/md.0000000000029904. 35945743 PMC9351861

[44] Z. Wang , P. Deurenberg , W. Wang , A. Pietrobelli , R. N. Baumgartner , and S. B. Heymsfield , “Hydration of Fat‐Free Body Mass: Review and Critique of a Classic Body‐Composition Constant,” American Journal of Clinical Nutrition 69, no. 5 (1999): 833–841, 10.1093/ajcn/69.5.833. 10232621

[45] D. W. Hill and J. M. Mihalek , “Calculation of a Conversion Factor for Estimating the Glycolytic Contribution in Exercise From Post‐Exercise Blood Lactate Concentration,” Frontiers in Physiology 14 (2024):1283327, 10.3389/fphys.2023.1283327. 38327682 PMC10847225

[46] T. Erselcan , F. Candan , S. Saruhan , and T. Ayca , “Comparison of Body Composition Analysis Methods in Clinical Routine,” Annals of Nutrition and Metabolism 44, no. 5–6 (2000): 243–248, 10.1159/000046691. 11146331

