# Supplementary figures and images for: The Oxygen Equivalent of Lactate Accumulation and Sex: Similar Work–Lactate Slopes in Men and Women Regardless of Body or Fat‐Free Mass Scaling
Source: FASEB Bioadv. 2026 Mar 17;8(3):e70097. doi: 10.1096/fba.2025-00330 (PMC13093647; doi:10.1096/fba.2025-00330)

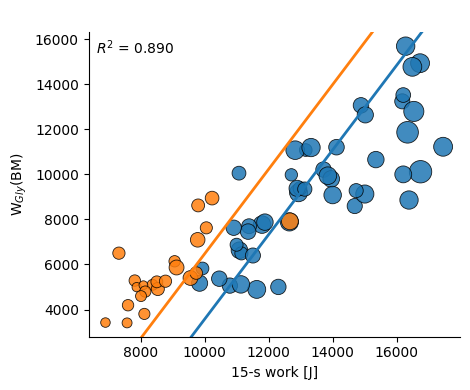

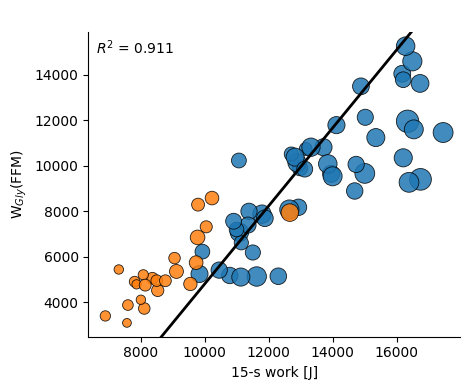

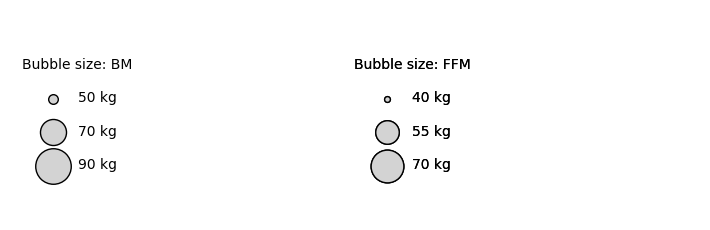

Supplement: Supplementary file 1 — Data S1: fba270097‐sup‐0001‐DataS1.docx. [file FBA2-8-e70097-s001.docx]
